# Cadherin Cytoplasmic Domains Inhibit the Cell Surface Localization of Endogenous E-Cadherin, Blocking Desmosome and Tight Junction Formation and Inducing Cell Dissociation

**DOI:** 10.1371/journal.pone.0105313

**Published:** 2014-08-14

**Authors:** Masayuki Ozawa, Wakako Kobayashi

**Affiliations:** Department of Biochemistry and Molecular Biology, Graduate School of Medical and Dental Sciences, Kagoshima University, Kagoshima, Japan; University of Birmingham, United Kingdom

## Abstract

The downregulation of E-cadherin function has fundamental consequences with respect to cancer progression, and occurs as part of the epithelial–mesenchymal transition (EMT). In this study, we show that the expression of the *Discosoma sp.* red fluorescent protein (DsRed)-tagged cadherin cytoplasmic domain in cells inhibited the cell surface localization of endogenous E-cadherin, leading to morphological changes, the inhibition of junctional assembly and cell dissociation. These changes were associated with increased cell migration, but were not accompanied by the down-regulation of epithelial markers and up-regulation of mesenchymal markers. Thus, these changes cannot be classified as EMT. The cadherin cytoplasmic domain interacted with β-catenin or plakoglobin, reducing the levels of β-catenin or plakoglobin associated with E-cadherin, and raising the possibility that β-catenin and plakoglobin sequestration by these constructs induced E-cadherin intracellular localization. Accordingly, a cytoplasmic domain construct bearing mutations that weakened the interactions with β-catenin or plakoglobin did not impair junction formation and adhesion, indicating that the interaction with β-catenin or plakoglobin was essential to the potential of the constructs. E-cadherin–α-catenin chimeras that did not require β-catenin or plakoglobin for their cell surface transport restored cell–cell adhesion and junction formation.

## Introduction

Cadherins comprise a large family of Ca^2+^-dependent cell–cell adhesion molecules. E-Cadherin, a prototypical member of this family, is a transmembrane protein that forms the adherens junction between epithelial cells. The cytoplasmic domain of E-cadherin interacts directly with β-catenin or plakoglobin. α-Catenin interacts with the cadherins indirectly via interactions with β-catenin or plakoglobin, and links the cadherin–catenin complex to the actin cytoskeleton through interactions with α-actinin, vinculin, formin, EPLIN (epithelial protein lost in neoplasm), and actin filaments [1]. p120 can interact with cadherins and regulates the steady-state levels and endocytosis of cadherins in cells [2], [3].

The loss of epithelial characteristics and the gain of a mesenchymal phenotype–a process referred to as the epithelial-to-mesenchymal transition (EMT)–is considered to be a hallmark of neoplastic transformation. A key initial step in EMT is the downregulation of E-cadherin, which at the transcriptional level is repressed by several factors: namely, ZEB1, ZEB2, Snail, Slug, and Twist [4]. The loss of E-cadherin is accompanied by the upregulation of mesenchymal markers, such as N-cadherin, fibronectin, and vimentin. Concomitant with these molecular changes, cells acquire a spindle-shaped mesenchymal morphology, and display enhanced migration and invasive properties [5].

*In vitro* studies using function-perturbing antibodies have indicated that E-cadherin-mediated adhesion is a necessary prerequisite for the formation of other cell junctions, including desmosomes and tight junctions [6]. An *in vivo* study employing the conditional inactivation of E-cadherin in stratifying epithelia showed that E-cadherin is required for tight junction, but not desmosome, formation [7], [8]. The upregulation of P-cadherin in the basal layer in combination with an increase in desmosomal cadherins may explain why E-cadherin is not essential for desmosome formation *in vivo*. Since a single cell type can express several different cadherins, the specific knockdown of E-cadherin may be accompanied by the increased expression of other cadherins, which compensate for the loss of E-cadherin [9].

Several cadherin functions have been elucidated using dominant-negative mutants. Pioneering work by Kintner [10] demonstrated that the exogenous expression in Xenopus embryos of mutant N-cadherins lacking the extracellular domain caused tissues to dissociate. This interesting phenomenon was ascribed to a dominant-negative effect of the introduced molecules on endogenous cadherin function. Other dominant-negative cadherins with similar structures have been described, and have also been shown to disrupt cell–cell adhesion and delay or reduce desmosome formation in keratinocyte cell lines [11], [12], [13]. Subsequent studies designed to elucidate the mechanism of action of the truncated dominant-negative cadherins revealed that: (1) the dominant-negative phenotype is not cadherin isotype–specific; (2) the dominant-negative cadherin must be able to associate with β-catenin and must be membrane-associated to be active; and (3) expression of the dominant-negative cadherin results in the down-regulation of endogenous cadherins by increasing their turnover rate [13], [14], [15].

It was recently shown that the expression of the dominant-negative protein induces EMT [16]. In another experiment, EMT was observed when E-cadherin expression was reduced by shRNA-mediated knockdown, but not by expression of the dominant-negative protein [17]. In the latter experiment, it was reported that the expression of the dominant-negative protein resulted in the loss of cell–cell contacts, but did not significantly induce the down-regulation of endogenous E-cadherin.

Here, we show that the expression of a *Discosoma sp.* red fluorescent protein (DsRed)-tagged cadherin cytoplasmic domain in MDCK cells inhibited the cell surface localization of endogenous E-cadherin, leading to morphological changes, the inhibition of assembly of desmosome and tight junction components, and a reduction in the mechanical integrity of the epithelial cell sheets. Thus, contrary to previous reports that the soluble cadherin cytoplasmic domains do not affect cadherin function, we showed that the cytoplasmic constructs exhibited dominant-negative activities. The observed morphological changes were not accompanied by the down-regulation of epithelial markers and the up-regulation of mesenchymal markers. Thus, these changes could not be classified as EMT. The constructs associated with β-catenin and plakoglobin, and reduced the level of β-catenin or plakoglobin associated with endogenous E-cadherin, raising the possibility that sequestration of β-catenin and plakoglobin by the constructs induced the intracellular localization of E-cadherin. The introduction of E-cadherin–α-catenin chimeras that did not require β-catenin or plakoglobin for their cell surface transport restored cell–cell adhesion and junction formation.

## Materials and Methods

### Ethics Statement

Experiments with recombinant DNA technology were performed in agreement with the guidelines of Kagoshima University Committee on recombinant DNA security.

### cDNA construction

The mammalian expression vectors containing hemagglutinin (HA)-tagged E-cadherin cDNA encoding either the wild-type (pC-EcadHA), or modified proteins (pC-EEAHA, pC-ESAHA, and pC-ELAHA), or HA-tagged N-cadherin (pC-NcadHA) were previously described [2], [18], [19]. These vectors were used as PCR templates for the production of the constructs used in this study. All PCR products were sequenced and cloned into expression vectors. The vectors containing the N-terminally DsRed-tagged and C-terminally FLAG-tagged E-cadherin cytoplasmic domain constructs (pC-DECT, pC-DECTEA, and pC-DECTSA), or the N-cadherin cytoplasmic domain (pC-NCT) were made as follows: cDNA encoding the cytoplasmic domains of E-cadherin or N-cadherin was obtained by PCR using the primer pairs CCTCGAGGGAGAACGGTGGTCAAAGA and ATCGTCGTCCTCGCCACCG or CCTCGAGGCCGGGATAAAGAACG and ATCGTCATCACCTCCACCATACA, respectively. The products were digested with Xho I and cloned into the Xho I and EcoR V sites of the pC-DsRedFLAG vector [20]. pC-DECTN and pC-DECTC, the chimeric constructs composed of DsRed and the N-terminal or C-terminal half of ECT, respectively, have been described [20]. cDNA encoding α-catenin residues 612–906 or residues 157–391 was amplified by PCR using the primer pairs GAGTTTATCGATGCTTCCCGC and ATCAATGCTGTCCATAGCTTTGAA or CCATCGATGTGGAAGATGGTATCTTGAA and ATCCTGTCTACGCAAGTCCC, respectively. The PCR products were digested with Cla I, and then cloned into the Cla I and EcoR V site of the pC-ELAHA vector, yielding pC-ELAαC and pCELAαM. An expression vector for DNCT under the control of the Tet-repressible transactivator (pU-DNCT) was constructed as follows: cDNA encoding DsRed, the N-cadherin cytoplasmic domain, and the FLAG tag in a pC-DNCT vector was amplified by PCR using the following primer pairs CCGGTCGCCACCATGGACAA and GCTCTAGACGCCCTTGTCGTC, digested with Xba I, and cloned into the EcoR I and Xba I site of the pUHD10-3 vector [21]. pCAGGSneo, pCAGGGSpur, and pCAGGShyg, which confer G418 resistance, puromycin resistance, and hygromycin resistance, respectively, have been described [22].

### Cells and Transfection

Cell culture conditions for the Type I Madin-Darby canine kidney (MDCK) cells have been described [22]. The type II MDCK cell clone (T23) [23], expressing the tet repressor, was provided by W. James Nelson of Stanford University. Cells were transfected using the calcium phosphate precipitation method, and selected using either G418 (1 mg/ml), puromycin (5 µg/ml), or hygromycin (300 µg/ml). Stable transfectants were identified by fluorescence microscopy and immunoblot, and were isolated as previously described [22]. At least three independent clones were selected for each construct to ensure that any observed effects were not due to phenotypic variability introduced by clonal selection.

### Antibodies

The following monoclonal antibodies were used to detect E-cadherin: DECMA-1, raised against the extracellular domain of E-cadherin (provided by Rolf Kemler of the Max-Planck Institute for Immunobiology); ECCD-2, recognizing the distinct extracellular domain of E-cadherin (Takara Bio Inc, Shiga, Japan); and C20820, a mAb detecting the cytoplasmic domain of E-cadherin (BD Biosciences, Lexington, KY). A rat mAb against HA (3F10) was purchased from Roche Molecular Biochemicals (Mannheim, Germany). A mouse mAb against FLAG (DYKDDDDK) was purchased from Wako Pure Chemical Industries, Ltd. (Osaka, Japan). Mouse mAbs recognizing N-cadherin, β-catenin, plakoglobin, and p120 were purchased from BD Biosciences, and a mAb detecting vinculin was obtained from Sigma-Aldrich Japan (Tokyo, Japan). A mouse mAb against vimentin, and rabbit antibodies targeting ZO-1, claudin 1, and occludin were purchased from Zymed Laboratories (South San Francisco, CA). A mouse mAb against desmoplakin was purchased from Progen Biotechnik GmbH (Heidelberg, Germany). All secondary antibodies were obtained from Jackson ImmunoResearch Laboratories, Inc. (West Grove, PA).

### Immunoprecipitation and immunoblotting

Immunoprecipitation and immunoblot analyses were carried out as described [19]. In brief, cells (2×10^6^) were lysed in a buffer (25 mM Tris-HCl buffer, pH 7.4, containing 1% Triton X-100, 2 mM EDTA, 10 mM sodium pyrophosphate, 10 mM NaF, 1 mM Na_3_VO_4_, 1 mM PMSF, 10 µg/ml leupeptin, and 25 µg/ml aprotinin). The proteins were collected with mAbs that had been preabsorbed to protein G–Sepharose.

### Fluorescence microscopy

Immunofluorescence labeling of cells was performed as described [22]. In brief, cells were fixed with 3% paraformaldehyde in PBS for 20 min at room temperature. Cells were permeabilized with 0.1% Triton X-100, and then incubated with primary and secondary antibodies. Cells were analyzed using an Olympus fluorescence microscope (Tokyo, Japan) equipped with a CD72 camera (Olympus) or a confocal laser scanning microscope (Zeiss LSM700).

### Dissociation assay

Cells were washed with PBS and then incubated for 2 h in DMEM supplemented with 10% FCS containing 2.4 U/ml of dispase (Gibco). Detached cells were subjected to mechanical stress by pipetting with a 1 ml pipette. The extent of cell dissociation was represented by the index Np/Nc, where Np and Nc are the total numbers of particles and cells per dish, respectively. To obtain Nc, the detached cells were incubated for 10 min in the presence of 5 mM EGTA, and then subjected to the mechanical stress.

### Wound healing assay

Cells were plated on 35 mm dishes and grown to confluency. Then, the cell monolayer was manually scratched with a pipette tip, washed with PBS, and incubated for 24 h. A phase contrast microscope was used to photograph the cells at 0 h and 24 h after performing the scratch.

### Invasion assay

Invasion was measured using BioCoat MatriGel Invasion Chambers (BD Biosciences) according to the manufacturer’s instructions. The lower chambers were filled with DME medium containing 5% FBS as a chemoattractant. The upper insert chambers were seeded with 2.5×10^4^ cells in serum-free DME medium. After incubation for 22 h at 37°C, the cells that penetrated through the Matrigel to the lower surface of the filters were stained, and then quantified by microscopy.

### Gene expression microarray and data analysis

Total RNA was prepared from DsRed+, DECT+, Snail+ MDCK cells using TRIzol Reagent (Invitrogen) and purified using SV Total RNA Isolation System (Promega) according to the manufacturer’s instructions. cRNA was amplified and labelled using a Quick Amp Labelling Kit (Agilent Technologies) and hybridized to a 44 K Agilent 60-mer oligomicroarray (Canine Oligo Microarray Kit; Agilent Technologies) according to the manufacturer’s instructions. The hybridized microarray slides were scanned using an Agilent scanner. The relative hybridization intensities and background hybridization values were calculated using Agilent Feature Extraction Software (version 9.5.1.1). Microarray data analysis was supported by Cell Innovator (Fukuoka, Japan).

## Results

### Expression of soluble cadherin cytoplasmic domains in MDCK cells disrupts cell–cell contacts

**Figure 1:**
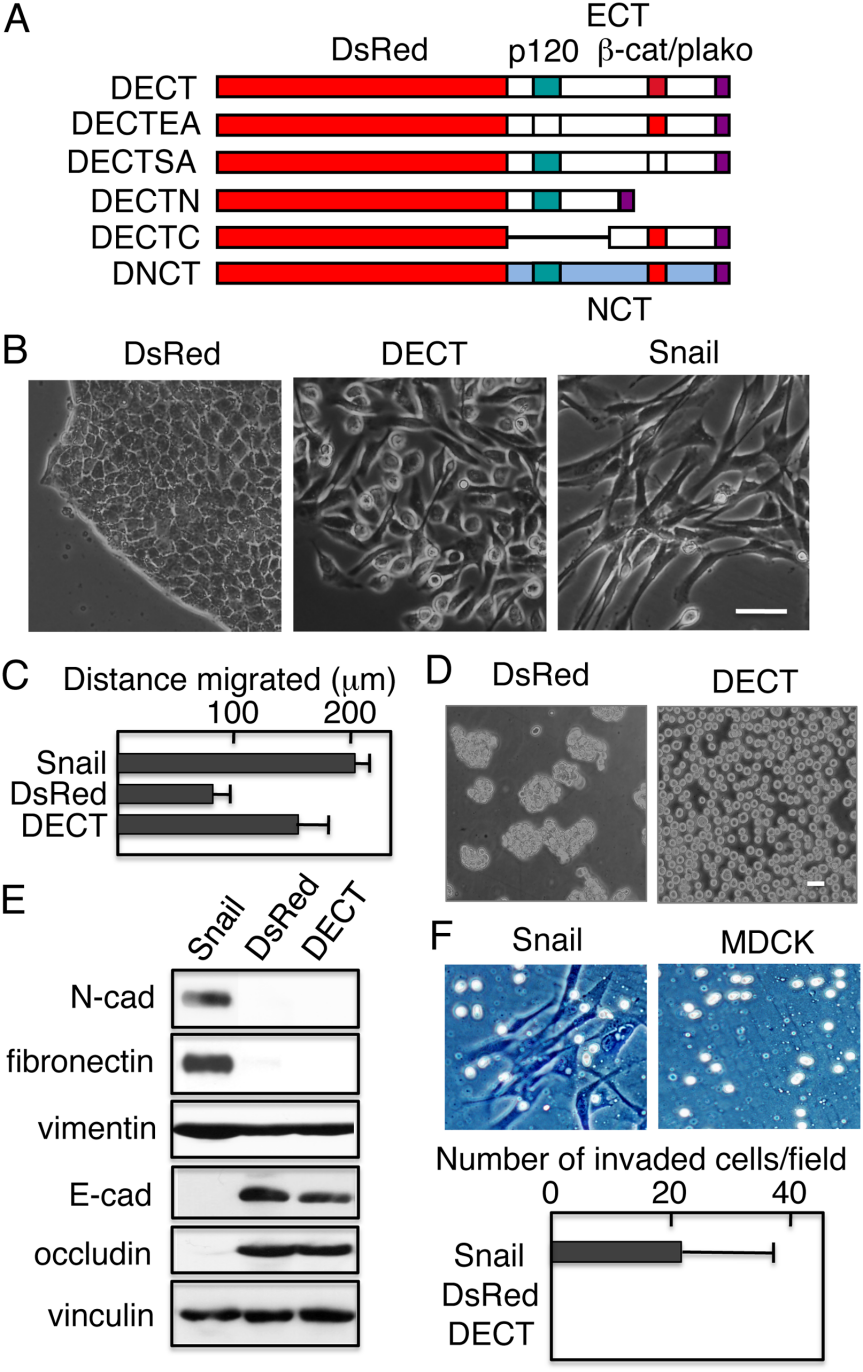
Expression of the DsRed-tagged E-cadherin cytoplasmic domain in MDCK cells disrupts cell–cell adhesion. (A) Schematic representation of DsRed-tagged cadherin cytoplasmic domain constructs. DECT is a DsRed-tagged wild-type E-cadherin cytoplasmic domain (ECT). The binding sites for p120 and β-catenin/plakoglobin (β-cat/plako) are shown. DECTEA is an ECT construct with alanine substitutions of the two conserved glutamic acid residues and a conserved aspartic acid residue (Glu-Glu-Asp) in the p120-binding site, which has been shown to eliminate the interaction with p120. DECTSA is an ECT construct with alanine substitutions of the conserved eight serine residues in the catenin-binding site, which has been shown to weaken the interaction with β-catenin. DECTN is a chimeric construct composed of DsRed and the N-terminal region of ECT containing the p120-catenin–binding site. DECTC is a chimera of DsRed and the C-terminal half of ECT containing the catenin–binding site. DNCT is an N-cadherin cytoplasmic domain (NCT) construct. The C-terminus of all constructs, including DsRed, is tagged with the FLAG epitope. (B) Morphology of MDCK cells expressing DsRed, DECT, and Snail. DECT+ and Snail+ cells lose cell–cell contacts. (C) The migration assay. While MDCK cells expressing Snail or DECT show enhanced migration, MDCK cells expressing DsRed do not. The results are represented as the mean ± SD of three independent experiments. (D) Dissociation assays. Cells were incubated with dispase and detached cells were subjected to mechanical stress by pipetting as described in Materials and Methods. (E) Immunoblot analysis revealed that up-regulation of fibronectin, N-cadherin, and vimentin and down-regulation of E-cadherin and occludin occurred in Snail+ cells but not in DECT+ cells. Vinculin was used as a loading control. (F) Invasion assays. Representative photographs of the cells that invaded (Snail+) and not invaded (parental MDCK) Matrigel (upper panels). The results are represented as the mean ± SD of three independent experiments. Bars, 25 µm.

We made the chimeric construct (DECT) comprising a fluorescent protein, DsRed, the cytoplasmic domain of E-cadherin (ECT), and a C-terminal FLAG tag (Fig. 1A), and expressed it in MDCK cells. A FLAG-tagged DsRed construct was used as a control. Including DsRed in the chimera facilitated the identification of cells expressing the protein. Cells expressing the control DsRed grew in monolayer cultures as epithelial clusters with a typical cobblestone morphology, whereas the expression of the DECT protein resulted in the loss of cell–cell contacts and in cell scattering (Fig. 1B). These changes were similar to those caused by the expression of Snail, a transcription factor known to induce EMT [22] (Fig. 1B). Concomitant with these morphological changes, DECT+ cells, like Snail+ cells, showed increased cell motility as compared to cells expressing DsRed as measured by wound-healing assays (Fig. 1C). Although DsRed cells migrated as cell sheets, DECT+ cells migrated as single cells (data not shown), raising the possibility that DECT+ cells had lost the integrity of the epithelial sheets.

The disruption of epithelial sheets containing DECT+ cells was confirmed by cell dissociation assays. DECT+ cells, but not DsRed cells, were dissociated into single cells after detachment from the dishes by dispase and subsequent mechanical dissociation by pipetting (Fig. 1D). Thus, DECT, but not DsRed, expression disrupted the mechanical integrity of the cell sheets.

The acquisition of these fibroblastic characteristics by the DECT+ cells suggested that the cells had undergone EMT. To determine whether the molecular alterations associated with EMT had occurred upon expression of DECT, we assessed the status of EMT markers in DECT+ cells using Snail+ cells as a positive control. Upon Snail-induced EMT, the expression of mesenchymal proteins, e.g., fibronectin, N-cadherin, and vimentin, was markedly upregulated (Fig. 1E). By contrast, none of the mesenchymal proteins was upregulated in DECT+ cells (Fig. 1E). Similarly, while the expression of E-cadherin and occludin, epithelial markers, was down-regulated in Snail+ cells, the expression of these proteins was maintained in DECT+ cells (Fig. 1E). Using an Agilent Whole Canine Genome microarray, we compared the gene expression profiles in MDCK cells expressing DsRed, DECT, or Snail. Although Snail expression changed the mRNA expression levels of fibronectin (>300-fold increase) and E-cadherin (>143-fold decrease), DECT expression did not change the mRNA levels of these proteins (data not shown). Thus, although Snail expression in MDCK cells induces an EMT, DECT expression caused inhibition of cell–cell adhesion and cell scattering without the additional molecular changes associated with the EMT program. The lack of down-regulation of endogenous E-cadherin in DECT+ cells was consistent with the previous observation that plasma membrane localization is required for the cytoplasmic domain of cadherins to down-regulate endogenous cadherins [13], [14], [15]. Consistent with the above observation that DECT did not induce EMT, DECT+ cells, unlike Snail+ cells, exhibited no invasive capacity in the MatriGel invasion assay (Fig. 1F).

**Figure 2:**
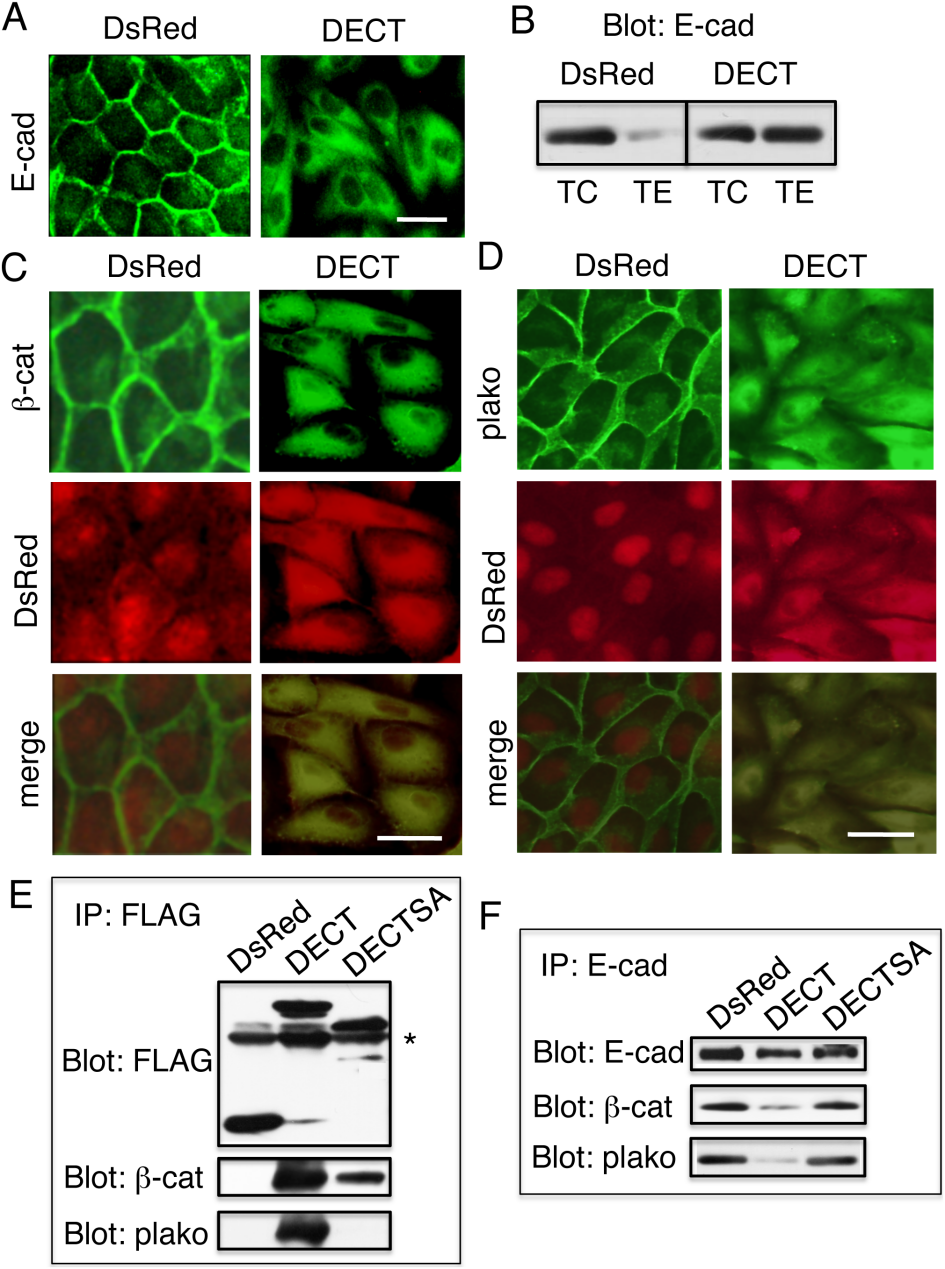
Expression of the DsRed-tagged E-cadherin cytoplasmic domain inhibits the cell surface localization of endogenous E-cadherin. (A) Immunofluorescence staining with DECMA-1, an antibody that recognizes the extracellular domain of E-cadherin, revealed that endogenous E-cadherin in DECT+ cells was localized intracellularly. (B) Tryptic digestion of cells with or without free Ca^2+^. Cells were incubated with 0.01% trypsin for 10 min at 37°C in the presence of 2 mM Ca^2+^ (TC) or 1 mM EGTA (TE). Then, immunostaining with an anti-E-cadherin mAb showed that a significant percentage of endogenous E-cadherin remained inside DECT+ cells. (C and D) Immunofluorescence staining with an anti-β-catenin (C) or anti-plakoglobin (D) antibodies revealed co-localization of β-catenin and plakoglobin with DECT. By contrast, β-catenin and plakoglobin did not co-localize with DsRed. Bars, 25 µm. (E) β-catenin and plakoglobin co-immunoprecipitated with DECT but not with DsRed, and (F) Reduced amounts of β-catenin and plakoglobin co-immunoprecipitated with endogenous E-cadherin in DECT+ cells as compared with DsRed cells. DECTSA, a DECT-derivative with alanine substitution of the conserved eight serine residues in the catenin-binding site, shows weakened interactions with β-catenin and plakoglobin (E) and did not significantly impair the complex formation of endogenous E-cadherin and β-catenin or plakoglobin (F). An asterisk in (E) indicates the position of the immunoglobin heavy chain.

Immunofluorescence staining using the E-cadherin monoclonal antibody, DECMA-1, which recognizes the extracellular part of the protein, revealed that endogenous E-cadherin did not exist at the surface of DECT+ cells (Fig. 2A). This was in contrast to cells expressing DsRed alone, which presented endogenous E-cadherin at their cell surface. Staining with a different anti-E-cadherin monoclonal antibody, ECCD-2, which also recognizes the extracellular part of the protein, yielded the same results (not shown). It is well known that cadherins at the cell surface resist tryptic digestion in the presence of Ca^2+^, but not in the absence of Ca^2+^. Cadherins localized in the intracellular compartments resists tryptic digestion even in the absence of Ca^2+^ [2]. Therefore, tryptic digestion of cells in the presence of 2 mM Ca^2+^ (TC) or 1 mM EGTA (TE), followed by immunoblot analysis with an anti-E-cadherin mAb has been used to quantify E-cadherin on the cell surface or inside the cells [18]. Consistent with its cell surface localization (Fig. 2A), ∼90% of endogenous E-cadherin on DsRed cells was digested following TE treatment (Fig. 2B). In contrast, ∼100% of endogenous E-cadhein in DECT+ cells was not digested after TE treatment (Fig. 2B). Thus, a significant percentage of endogenous E-cadherin remained inside DECT+ cells. As a control, the apical membrane marker protein gp135 [24] was detected at the cell surface (data not shown), indicating that its transport to the cell surface was not affected by DECT.

Immunofluorescence staining of DECT+ cells revealed that β-catenin did not exist on the cell surface membrane, but rather that it co-localized with DECT in intracellular compartments (Fig. 2C). Although DsRed was detected in the intracellular compartment of DsRed cells, it did not change the distribution of β-catenin. Likewise, the co-localization of plakoglobin with DECT in the intracellular compartment was observed in DECT+ cells (Fig. 2D). The co-localization of β-catenin and plakoglobin with DECT suggested that they formed a complex. Immunoprecipitation of DECT or DsRed using the anti-FLAG antibody, followed by immunoblotting with the anti-β-catenin or anti-plakoglobin antibody, revealed that β-catenin and plakoglobin formed a complex with DECT but not with DsRed (Fig. 2E). Immunoblot detection of β-catenin or plakoglobin co-immunoprecipitated with endogenous E-cadherin collected by antibodies raised to the extracellular part of E-cadherin revealed that reduced amounts of β-catenin and plakoglobin were co-immunoprecipitated with endogenous E-cadherin in DECT+ cells as compared to DsRed cells (Fig. 2F). The results suggested the possibility that DECT competed with endogenous E-cadherin for β-catenin and plakoglobin binding, and reduced the amounts of β-catenin and plakoglobin associated with endogenous E-cadherin. Confocal images revealed a possible colocalization of DECT with β-catenin or plakoglobin in the nucleus (Fig. S1), raising the possibility that they may alter transcription as described previously [25].

### The ability to interact with β-catenin and plakoglobin is essential for the potential of the cytoplasmic domain

Alanine substitution of the conserved eight serine residues in the catenin-binding site of E-cadherin has been shown to weaken the interaction with catenins [19], [26]. To determine the requirement for catenin-binding, the same serine to alanine substitutions were introduced into DECT, yielding DECTSA (Fig. 1A). p120 is another binding partner of the cadherin cytoplasmic domain. Alanine substitution of the two conserved glutamic acid residues and the conserved aspartic acid residue (Glu-Glu-Asp) in the p120-binding site of E-cadherin has been shown to eliminate its interaction with p120 [27]. We introduced the same substitutions into DECT to yield a p120-uncoupled construct (DECTEA; Fig. 1A). To determine that the cytoplasmic domain activity was specific for E-cadherin, we made a construct composed of DsRed and the cytoplasmic domain of N-cadherin (DNCT; Fig. 1A).

**Figure 3:**
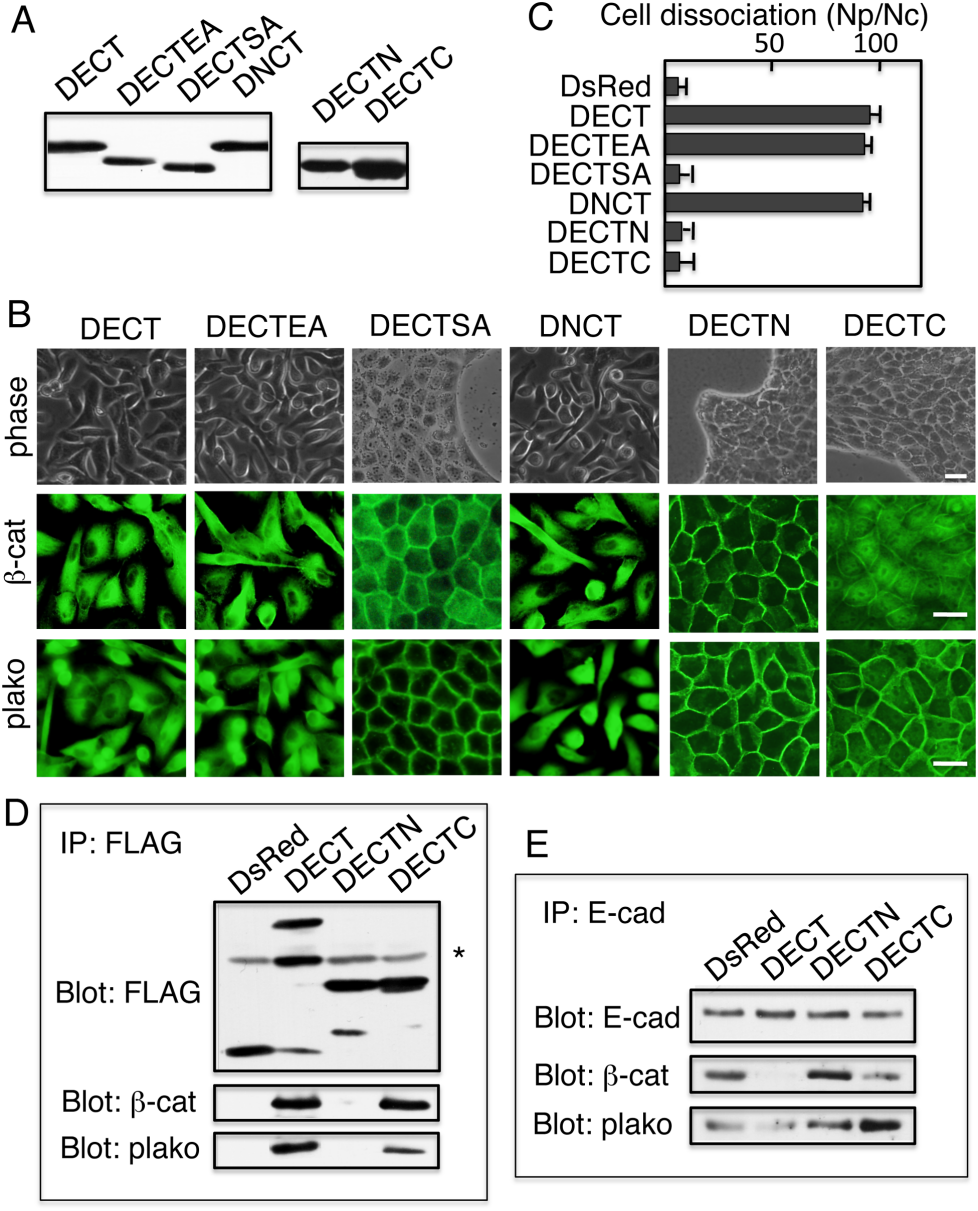
The ability to interact with β-catenin/plakoglobin is essential to the potential of the cytoplasmic domains. (A) Immunoblot detection of the constructs with anti-FLAG antibodies. DECTSA and DECTEA migrated faster than DECT and DNCT. DECTN and DECTC showed similar mobility. (B) Phase contrast (upper panels), β-catenin (middle panels, β-cat), and plakoglobin immunofluorescence (lower panels, plako) images of cells expressing DECT, DECTSA, DECTEA, DNCT, and DECTN, and DECTC. Expression of DECT, DECTEA, and DNCT disrupted cell–cell contacts and induced the intracellular localization of β-catenin and plakoglobin. Expression of DECTC also induced the intracellular localization of β-catenin, but did not affect cell-cell contacts and plakoglobin remained associated with the plasma membrane. Expression of DECTSA did not affect cell–cell contacts and significant amounts of β-catenin remained associated with the plasma membrane. Bars, 25 µm. (C) Quantification of cell dissociation assays. The extent of cell dissociation was represented by the index Np/Nc, where Np and Nc are the total numbers of particles and cells per dish, respectively. Expression of DECT, DECTEA, and DNCT disrupts the mechanical integrity of cell sheets. Cells expressing DsRed, DECTSA, DECTN, and DECTC retain the mechanical integrity of their cell sheets. The results are represented as the mean ± SD of three independent experiments. (D) β-catenin co-immunoprecipitated with DECT or DECTC but not with DsRed or DECTN. Plakoglobin also interacted with DECT and DECTC. However, reduced amounts of plakoglobin co-immunoprecipitated with DECTC, indicating that DECTC shows weakened interactions compared with DECT. An asterisk in indicates the position of the immunoglobin heavy chain. (E) Reduced amounts of β-catenin and plakoglobin co-immunoprecipitated with endogenous E-cadherin in DECT+ cells as compared with DsRed+ or DECTN+ cells. DECTC did not impair the complex formation of endogenous E-cadherin and plakoglobin. Quantification of images revealed increased amounts of plakoglobin co-immunoprecipitated with E-cadherin in DECTC+ cells (see Table 1).

Stable transfectants expressing these proteins were isolated and confirmed by immunoblot analysis (Fig. 3A). Like DECT, expression of DECTEA and DNCT, but not DECTSA, disrupted cell–cell contacts (Fig. 3B, upper panels). Note that cells expressing DECT, DECTEA, or DNCT never made dense cell cultures. Expression of DECTEA and DNCT, but not DECTSA, changed the intracellular localization of β-catenin and plakoglobin (Fig. 3B, middle and lower panels). Disruption of the integrity of epithelial cell sheets by the expression of DECTEA and DNCT, but not by the expression of DECTSA, was confirmed by dissociation assays. DECTEA+ and DNCT+ cell sheets were dissociated, but DECTSA+ cell sheets remained intact (Figs. 3C). Like MDCK cells, the expression of DNCT, but not DsRed, in HaCaT cells, the immortalized, nontumorigenic cell line, induced morphological changes and weakened the integrity of the epithelial sheets (data not shown). Reduced amounts of β-catenin and plakoglobin co-immunoprecipitated with DECTSA as compared to DECT (Fig. 2E). The expression of DECTSA did not seem to impair the association of endogenous E-cadherin with β-catenin and plakoglobin, because the amount of β-catenin and plakoglobin that co-immunoprecipitated with endogenous E-cadherin was similar between DECTSA+ and DsRed cells (Fig. 2F). Together, these data show that the ability to interact with β-catenin and plakoglobin was essential for the potential of the cytoplasmic domain.

Previous experiments using E-cadherin deletion proteins revealed that the C-terminal half of the cytoplasmic domain is enough for binding of catenins [28]. To determine the ability to interact with β-catenin and plakoglobin is sufficient for the potential of the cytoplasmic domains, two additional fusion proteins were constructed. The N-terminal half of the cadherin cytoplasmic domain, which carries the p120–binding site, and the C-terminal half of the domain, which encodes the β-catenin and plakoglobin–binding site (Fig. 1A), were independently fused to DsRed, generating two chimeras: the N-terminal (DECTN) and C-terminal (DECTC) fusion proteins (Fig. 1A). The cytoplasmic domain of cadherins or even the C-terminal half of the domain has been shown to sequester β-catenin and prevents it from binding to LEF/TCF, thus inhibits β-catenin–dependent LEF/TCF transcriptional activity [29]. Consistent with the previous reports, although expression of the N-terminal chimera (DECTN) in MDCK cells did not change the membrane distribution of β-catenin, expression of the C-terminal chimera (DECTC) changed the β-catenin distribution, β-catenin was exclusively detected the cytoplasm (Fig. 3B middle panels). The distribution of plakoglobin, however, was not affected by the DECTC expression (Fig. 3B lower panels). In these cells, E-cadherin detected by DECMA-1 was found on the cell surface (data not shown). More importantly, the cells show normal epithelial morphology (Fig. 3B upper panel) and show integrity of epithelial sheets as measured by dissociation assay (Fig. 3C). These results raised a possibility that although DECT and DECTC bind to β-catenin at the similar levels, the binding level of DECTC to plakoglobin is not similar to that of DECT. Thus, DECTC cannot sequester plakoglobin from the endogenous E-cadherin. To confirm this, DECT or DECTC were collected using anti-FLAG antibody and then subjected to immunoblot with anti-β-catenin and anti-plakoglobin antibodies. As expected, both DECT and DECTC collected the similar amounts of β-catenin from the cells expressing respective constructs (Fig. 3D). The amount of plakoglobin collected by DECTC is much lower than that collected by DECT. Thus, the ability of DECTC to bind plakoglobin is not high as DECT. DsRed and DECTN did not bind to β-catenin or plakoglobin. Consistent with these observations, the amounts of β-catenin bound to endogenous E-cadherin were decreased in DECTC+ cells (Fig. 3E and Table 1). Importantly, the amounts of plakoglobin bound to endogenous E-cadherin did not decrease but rather increased in DECTC+ cells (Fig. 3E and Table 1). Together, these data strongly suggested that plakoglobin was not significantly depleted by DECTC expression and plakoglobin compensated the shortage of β-catenin for endogenous E-cadherin in DECTC+ cells. Therefore DECTC+ cells show the surface membrane localization of endogenous E-cadherin, normal epithelial morphology, and show integrity of epithelial sheets.

**Table 1:** Relative amounts of catenins co-precipitated with endogenous E-cadherin isolated from MDCK cells expressing different constructs.

	DsRed	DECT	DECTN	DECTC
β-catenin	100	4±2	102±37	24±21
plakoglobin	100	33±18	100±57	187±80

**Figure 4:**
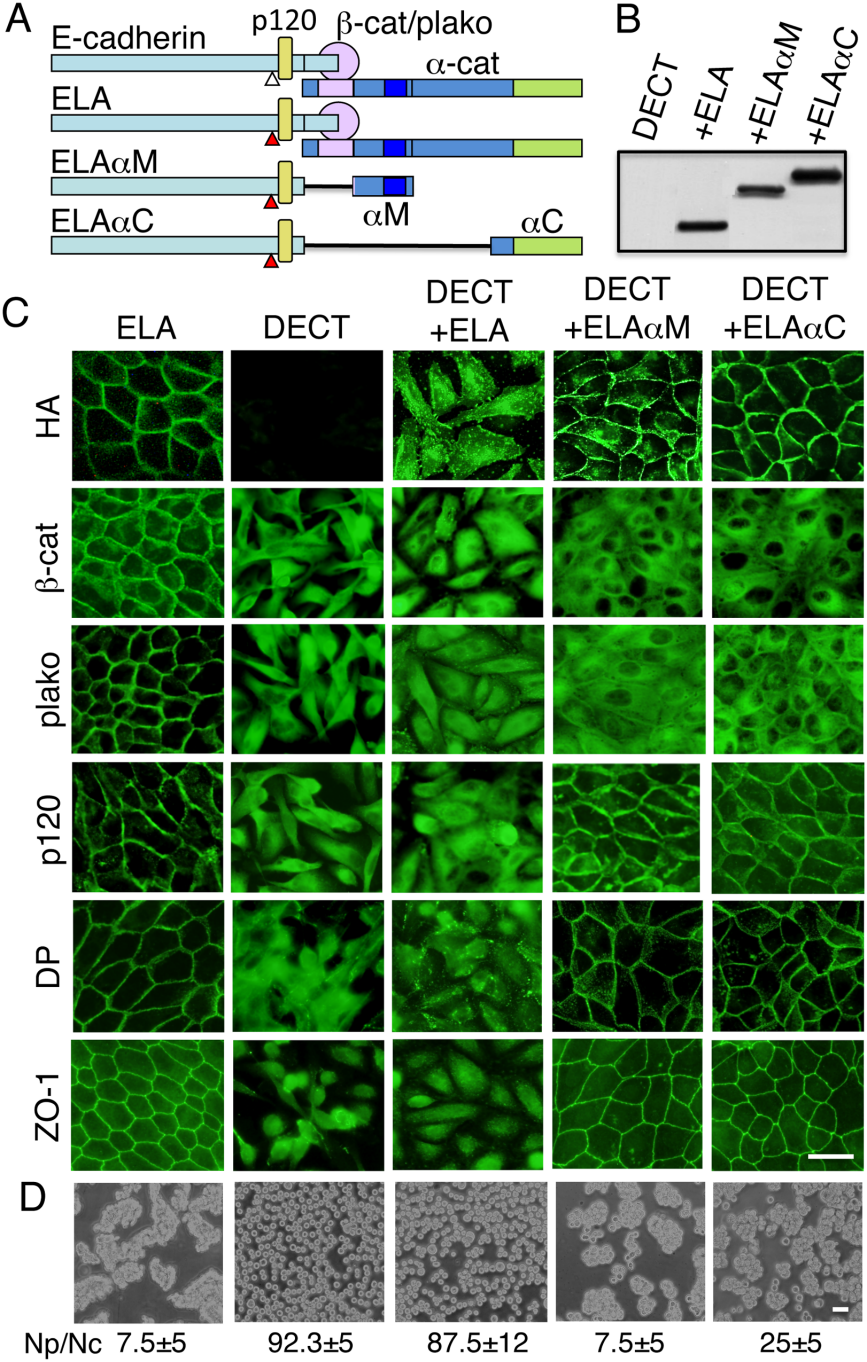
E-cadherin–α-catenin chimeric molecules restore cell–cell adhesion and junctional assembly. (A) Schematic representation of E-cadherin and its three derivatives. E-cadherin associates with catenins (α-cat and β-cat/plako) and p120. ELA is a mutant E-cadherin in which two leucine residues at positions 587 and 588, which are close to the p120-binding site, were substituted with two alanine residues. This substitution improves the cell surface localization of E-cadherin. ELAαM and ELAαC are ELA-α-catenin chimeric proteins consisting of (a) the entire extracellular and transmembrane domains of E-cadherin as well as the first 80 amino acids of its cytoplasmic domain, excluding the region required for β-catenin or plakoglobin-binding, and (b) α-catenin regions encompassing either amino acids 157–381 or 612–906, which include the domains necessary for association with formin/vinculin or ZO-1/actin, respectively, but not the domain essential for association with β-catenin (α-catenin residues 48–163). Thus, ELAαM and ELAαC could not associate with β-catenin but could still interact with p120. All constructs were tagged with HA. (B) Immunoblot detection of ELA, ELAαM, and ELAαC chimeras expressed in DECT+ cells. Cell lysates prepared from DECT+ cells and DECT+ cells expressing ELA (+ELA), ELAαM (+ELAαM), or ELAαC (+ELAαC) were analyzed. Blots were stained with anti-HA antibodies. (C) Immunofluorescence staining of MDCK cells expressing ELA, DECT, or the indicated combinations of proteins: DECT and ELA (DECT+ELA), DECT and the ELAαM chimera (DECT+ELAαM), or DECT and the ELAαC chimera (DECT+ELAαC). The expression of DECT in MDCK cells induced the intracellular accumulation of not only β-catenin (β-cat) and plakoglobin (plako), but also p120, desmoplakin (DP), and ZO-1. Significant amounts of ELAαM and ELAαC, but not ELA, were observed at the cell surface as detected by anti-HA. The expression of ELAαM or ELAαC, but not ELA, in DECT+ cells induced the redistribution of p120, desmoplakin (DP), and ZO-1 to the cell surface. β-catenin and plakoglobin in the same cells remained in the cytoplasm. (D) Dissociation assays. Quantified data were shown under each panel. The results are represented as the mean ± SD of three independent experiments. Expression of ELAαM and ELAαC in DECT+ cells restored the mechanical integrity of cell sheets. Bars, 25 µm.

β-catenin and plakoglobin co-precipitated with endogenous E-cadherin as described in Fig. 4E and two another experiments were quantified using NIH Image and expressed as a percentage of the proteins isolated from cells expressing DsRed. Values are the mean ± the S.E. for triplicate determinations.

### E-cadherin–α-catenin chimeric molecules that do not require β-catenin and plakoglobin for their cell surface transport restore cell–cell adhesion and junction assembly

To show that the disruption of cell–cell junction biogenesis was due to a depletion in β-catenin and plakoglobin, which is required for the correct localization of E-cadherin, we performed rescue experiments using E-cadherin–α-catenin chimeric molecules (Fig. 4A). The chimera, composed of C-terminal truncated E-cadherin and the C-terminal one-third of the α-catenin polypeptide (residues 612–906, EαC), when expressed in L cells, is transported to the cell surface and is active in aggregation assays [30]. To improve the cell surface expression of the chimera in MDCK cells, two leucine residues (at positions 587 and 588) in the juxtamembrane cytoplasmic domain, which are required for the efficient endocytosis of E-cadherin [2] and the intracellular retention of β-catenin-uncoupled E-cadherin [18], were substituted with two alanine residues, yielding ELAαC (Fig. 4A). As a control, we used E-cadherin with the same leucine to alanine (LA) substitutions (ELA; Fig. 4A). The second chimera (ELAαM) contains α-catenin regions of amino acids 157–381. Like ELAαC, when ELAαM is expressed in L cells, it is transported to the cell surface and is active in aggregation assays (Ozawa, unpublished observations).

Expression vectors for these constructs, ELA, ELAαM, and ELAαC, were introduced into DECT+ cells, established by selection with hygromycin, and stable double transfectants were isolated after selection with G418, followed by immunostaining and immunoblotting with anti-HA (Fig. 4B). ELA expressed in MDCK cells was localized exclusively to the cell surface, but the same molecule expressed in DECT+ cells was detected both in intracellular compartments and at the cell surface (Fig. 4C). The unavailability of β-catenin and plakoglobin to complex with ELA, despite the LA substitution, may have been responsible for the intracellular localization of the protein. The E-cadherin chimeras with α-catenin (ELAαM and ELAαC) expressed in DECT+ cells were detected at the cell surface (Fig. 4C). These proteins could not interact with β-catenin and plakoglobin because of the deletion of the catenin-binding sites; therefore, they did not significantly change the distribution of β-catenin and plakoglobin (Fig. 4C), although small amount of plakoglobin was localized to the cell surface. By contrast, the constructs retained the ability to interact with p120, and thus re-localized significant amounts of p120 to the cell surface (Fig. 4C).

The easy mechanical dissociation of DECT+ cells suggested that the expression of DECT affected the assembly of adherens junctions and other junctional complexes, e.g., desmosomes and tight junctions. Consistent with this idea, the expression of DECT in MDCK cells induced the intracellular localization of desmoplakin, a desmoglein-associated desmosomal protein, and ZO-1, a tight junction component (Fig. 4C).

The expression of ELAαM and ELAαC chimeras in DECT+ cells restored the mechanical integrity of cell sheets because cells expressing these proteins were resistant to mechanical dissociation (Figs. 4D). Consistent with the mechanical integrity of DECT+ cells expressing ELAαM and ELAαC, desmoplakin was detected on the plasma membrane of the cells expressing these proteins (Fig. 4B). ZO-1 was also detected on the plasma membrane of DECT+ cells expressing ELAαM and ELAαC (Fig. 4B). These results suggested that desmosomes and tight junctions were established by expressing ELAαM and ELAαC, despite the presence of DECT, which sequestered β-catenin and plakoglobin and prevented the cell surface localization of endogenous E-cadherin or exogenously introduced ELA protein.

### The action of DNCT is reversible

**Figure 5:**
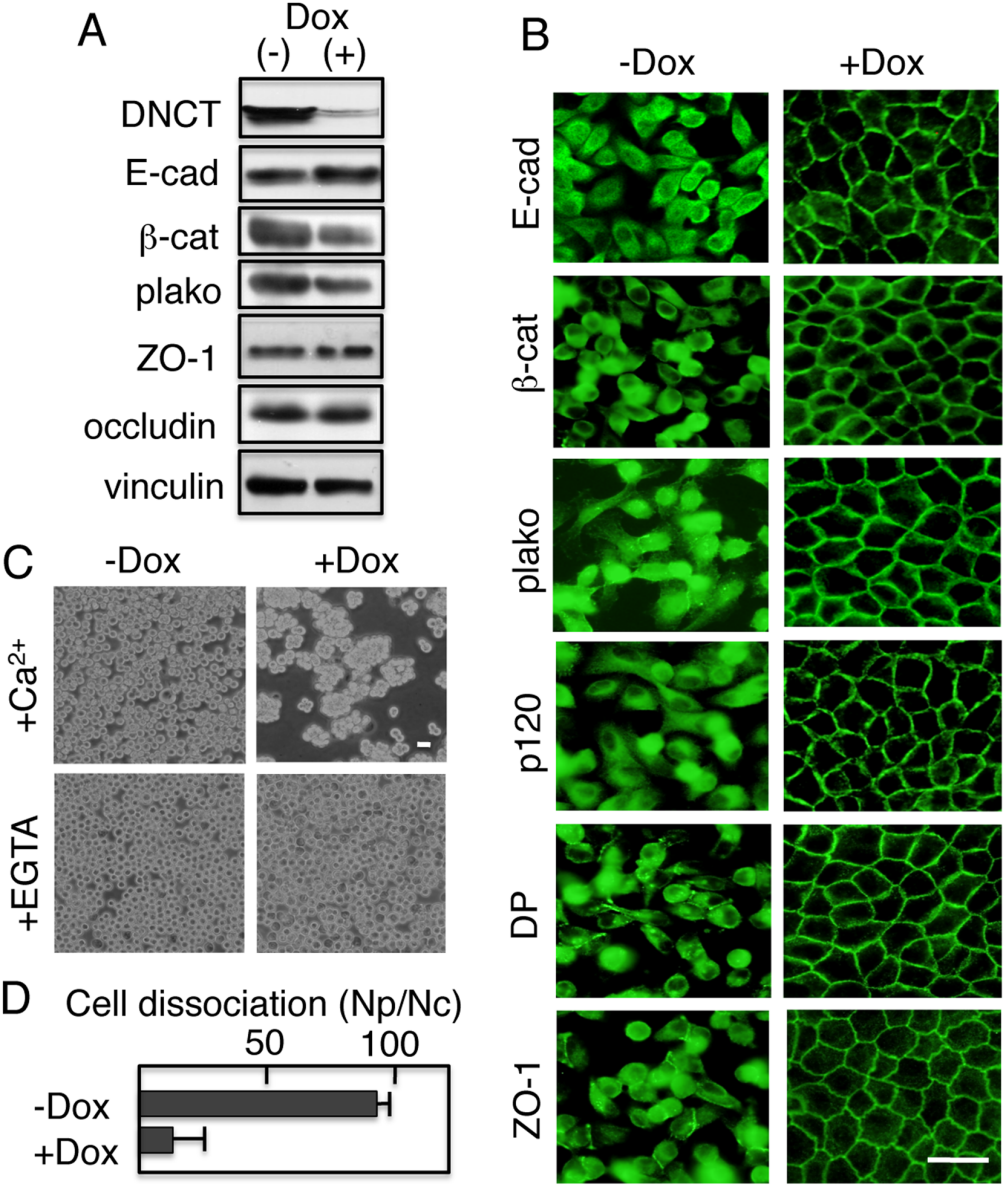
Reversibility of the cadherin cytoplasmic domain activity. (A) Dox-repressible expression of DNCT in MDCK cells. An MDCK derivative (T23), expressing the tet repressor, was transfected with an expression vector encoding DNCT under the control of the tet promoter. Clones showing tet-repressible expression of DNCT were isolated. The cells of a representative clone were cultured for 4 days with (+) or without (−) Dox, and subjected to immunoblot analysis with the indicated antibodies. DNCT was detected with an anti-FLAG antibody. The addition of Dox repressed DNCT expression and induced a slight increase in E-cadherin expression, but did not affect the expression of other proteins. Vinculin was used as a loading control. (B) Expression of DNCT inhibited the cell surface localization of endogenous E-cadherin (E-cad); its associated proteins, β-catenin (β-cat), plakoglobin (plako), and p120 (p120); the desmosomal protein, desmoplakin (DP); and the tight junction protein, ZO-1. The addition of Dox induced the cell surface localization of these components. (C) Dissociation assays. Cells were cultured in the presence (+) or the absence of Dox (−) for 4 days, and then subjected to the assay. Cells treated with Dox retained the mechanical integrity of their cell sheets, but untreated cells did not. Detached cell sheets became dissociated in the presence of EGTA. Thus, the mechanical integrity depended on the presence of Ca^2+^ in the medium. (D) Quantification of cell dissociation assays. The results are represented as the mean ± SD of three independent experiments. Bars, 25 µm.

To explore the reversibility of the cadherin cytoplasmic domain activity, we expressed DNCT under the control of the Tet-repressible transactivator. In the presence of either Tet or Dox, the expression of exogenous protein is repressed [21]. A DNCT expression vector was introduced into T23 cells, an MDCK cell clone expressing the tet repressor [23], and stable transfectants were isolated. Immunoblot analysis of DNCT+ T23 cells cultured for 4 days with or without doxycycline showed that DNCT was detected in protein extracts from cells cultured without Dox, but was significantly reduced (∼17%) in extracts from cells cultured with Dox (Fig. 5A). We examined whether DNCT expression affected the levels of endogenous E-cadherin and other junctional proteins by comparing their amounts in lysates from cells cultured with or without Dox. DNCT expression slightly decreased the levels of endogenous E-cadherin (∼93%), and increased the levels of β-catenin (∼123%) and plakoglobin (∼122%). DNCT expression did not significantly affect the levels of other junctional components, including ZO-1 and occludin (Fig. 5A). The expression of DNCT, however, did affect the distribution of E-cadherin and β-catenin and plakoglobin in the Tet-repressible system (Fig. 5B). The expression of DNCT in T23 MDCK cells induced the intracellular localization of E-cadherin, β-catenin, plakoglobin, p120, desmoplakin, and ZO-1 (Fig. 5B). Culturing cells in the presence of Dox completely reversed the intracellular accumulation of these components, which were subsequently detected at the plasma membrane (Fig. 5B). Thus, the action of DNCT is reversible. Removal of Dox from the culture media and culturing for 5 days in the absence Dox again induced the intracellular accumulation of the components (data not shown).

When the cells cultured for 4 days with or without Dox were subjected to dissociation assays, the cells cultured without Dox were dissociated but cells cultured in the presence of Dox were resistant to mechanical dissociation (Figs. 5C and D). Therefore, in the presence of Dox, the epithelial sheet integrity was functionally reestablished by down-regulating the expression of DNCT. The action of dispase requires Ca^2+^. Therefore, the Ca^2+^-dependency of the sheet integrity was assessed as follows. Cells were first detached from the dishes by dispase in the presence of Ca^2+^. Then, the detached cells were incubated in the presence of EGTA for an additional 10 min, and subjected to mechanical dissociation. When the detached cells were incubated with EGTA to remove Ca^2+^ before mechanical dissociation, the cells were completely dissociated (Fig. 5C). Thus, the stability to mechanical dissociation was dependent on Ca^2+^.

## Discussion

Downregulation of E-cadherin expression or functional perturbations of E-cadherin–catenin complexes frequently occur during oncogenesis, and have been casually linked to the progression of adenoma to invasive carcinoma. The molecular consequences of E-cadherin downregulation/perturbation during tumor progression have been extensively studied, but are still not completely understood [31]. Mutant cadherins lacking the extracellular domain have been used to elucidate cadherin functions because their expression leads to the loss of cell–cell contacts; therefore, these constructs act as dominant-negative proteins. However, their activity seems to be rather limited because their expression does not prevent desomosome or tight junction assembly [32], or it only delays/reduces the formation of desmosomes [11], [12], [13]. Furthermore, their mode of action remains elusive. These proteins down-regulate endogenous cadherins by increasing their turnover rate. They must be localized to the membranes to be active; in fact, soluble forms of the cadherin cytoplasmic domains have been reported to be inactive as inhibitors of endogenous E-cadherin [14], [15]. Contrary to these previous studies, we found that the soluble forms of the cadherin cytoplasmic domains, by sequestering β-catenin and plakoglobin from endogenous E-cadherin, inhibit the transport of endogenous E-cadherin to the cell surface and induce cell dissociation. In addition, they inhibit the formation of desmosomes and tight junctions. At present we do not know the reason for this discrepancy.

Since the amino acid substitutions introduced into the cytoplasmic domain of E-cadherin weakened its interaction with β-catenin and plakoglobin and abrogated its activities as a dominant-negative protein, the amount of the cytoplasmic domain produced may be critical to the experimental outcome. In the previous experiments, 1) the cytoplasmic domains of E-cadherin or N-cadherin [14], or 2) a fusion protein of the E-cadherin cytoplasmic domain with glutathione S-transferase [15] were used. The differences in the stability between our fusion proteins and other constructs may explain the disparate results. An N-cadherin cytoplasmic domain fusion protein with GFP has been described [29]. This chimera has been successfully used as an inhibitor of β-catenin-dependent Wnt signaling pathways because it can sequester β-catenin from LEF-1/Tcf transcription factors [29]. In these experiments, CHO cells and SW480 cells were used and stable transfectants were isolated. CHO cells have no endogenous cadherins, and thus serve as excellent hosts for exogenous cadherin molecules [33]. Therefore, CHO cells are not suitable cells for the analysis of dominant-negative mutant cadherin activity. SW480 cells are a colorectal cancer cell line with a mutated adenomatous polyposis coli (APC) gene. Since APC is involved in the GSK3β-mediated phosphorylation and subsequent ubiquitin-dependent degradation of β-catenin, SW480 cells display increased levels of β-catenin [34]. Furthermore, β-catenin binds the cytoplasmic domain of cadherin with higher affinity than LEF-1 [35]. Therefore, it is possible that the amount of the N-cadherin cytoplasmic domain-GFP fusion protein is not sufficient to prevent the transport of cadherins in these cells, although it is enough to block LEF-1/Tcf-dependent transcription. In the subsequent experiments using the yeast two-hybrid system and transient expression of GFP-tagged proteins in mammalian cells, these authors identified cadherin sequences that inhibit β-catenin signaling [36]. They found that expression of the GFP-tagged entire cytoplasmic domain of DE-cadherin (*Drosophila* E-cadherin) in MDCK cells resulted in partial disruption of adherens junctions and the accumulation of β-catenin in the cytoplasm and the nuclei. Expression of the shorter (30 amino acid) cadherin tail fragment, which effectively inhibited β-catenin-mediated signaling, in MDCK cells resulted in the accumulation and diffuse distribution of β-catenin but without a detectable effect on its organization in adherens junctions. These authors, however, did not examine the interaction of the fragments with plakoglobin. As shown in the present study, the shorter E-cadherin cytoplasmic domain (DECTC) having β-catenin-binding ability showed weaker binding to plakoglobin than the entire cytoplasmic domain and loses the ability to disrupt junctions. Another E-cadherin cytoplasmic domain chimera fused to βTrCP ubiquitin–protein ligase was expressed in DLD1 cells [37]. DLD1 cells are also a colorectal cancer cell line with another APC mutation [38]. Again, the APC mutation in this cell line may explain why the authors found an attenuation of β-catenin signaling, but no effect on adhesion junctions in cells expressing the chimera.

It has been shown that the expression of a dominant-negative mutant of E-cadherin (Ec1WVM, a mutant E-cadherin protein with a nonfunctional extracellular domain) in A431 cells caused EMT [16]. However, in the present study we showed that the expression of the soluble cadherin cytoplasmic domain in normal MDCK epithelial cells did not induce EMT. Immunoblot analysis of the cells expressing the soluble cadherin cytoplasmic domains revealed that the changes induced by these proteins could not be classified as EMT, because no down-regulation of epithelial markers (E-cadherin and occludin) and no up-regulation of mesenchymal markers (fibronectin, N-cadherin, and vimentin) took place. Consistent with our observation, it has been shown that an extracellular domain–deleted dominant-negative E-cadherin expressed in immortalized human breast epithelial cells did not induce EMT [17]. Thus, it seems that although the expression of dominant-negative cadherins in normal cells does not induce EMT, in cancer cells it results in EMT. Therefore, it is possible that the loss of cadherin function could significantly contribute to carcinogenesis and metastasis if it occurred within the context of other changes, such as growth stimulatory mutations.

The cell–cell adhesion molecule E-cadherin is localized to the basolateral membrane of polarized epithelial cells. Little is known, however, about mechanisms regulating the intracellular trafficking of E-cadherin [39]. Previous studies indicated that newly synthesized E-cadherin binds to β-catenin soon after its synthesis, and that α-catenin binds to the complex when E-cadherin arrives at the cell surface [40], [41]. In addition, mutant E-cadherin deficient in β-catenin and plakoglobin-binding localized to intracellular compartments [18], [42]. Consistent with these observations, the targeting of E-cadherin to the plasma membrane was blocked in β-catenin and plakoglobin double-null cells [43]. Thus, E-cadherin–β-catenin or E-cadherin-plakoglobin complex formation seems to be a prerequisite for the efficient transport of E-cadherin to the cell surface. Our observation that the cadherin cytoplasmic domain constructs had the ability to deplete β-catenin and prevent cell surface localization of endogenous E-cadherin is consistent with this idea.

The defects in the cell junctions of DECT+ cells were rescued by the expression of E-cadherin–α-catenin chimeras (ELAαM and ELAαC). Immunofluorescence staining of the rescued cells revealed that the tight junction and desmosomal components were properly assembled. Furthermore, the cell sheets resisted the mechanical force inflicted in the dissociation assay. We do not know how these chimeric proteins rescued the junctional assembly defect in DECT+ cells. Although ELAαM and ELAαC could not interact with β-catenin and plakoglobin, they were efficiently transported to the sites of cell–cell contact. α-Catenin is a protein with multiple domains that interacts with a number of proteins, including actin and actin-binding proteins–actin (α-catenin residues 685–883) [44], ZO-1 (α-catenin residues 631–906) [45], vinculin (α-catenin residues 326–509) [46], formin (α-catenin residues 300–500) [47], and α-actinin (α-catenin residues 325–394) [48]. Thus, the ELAαC chimera (containing the α-catenin residues 612–906) or the ELAαM chimera (containing the α-catenin residues 157–381) may interact with one of these proteins and effect transport to junctional sites. It has been shown that the delivery of cadherins and catenins to the cell surface requires cortical actin filaments [49].

Previous studies have suggested that the actin cytoskeleton is involved in desmosomal and tight junction assembly, which are sensitive to various actin-disrupting drugs, e.g., cytochalasin D [50], [51]. It has been shown that the interactions of E-cadherin with ZO-1 or vinculin through α-catenin play fundamental roles in the assembly of these structures [45], [46]. Interestingly, both vinculin and ZO-1 are actin-binding proteins. Since ELAαM and ELAαC have the ability to interact with these cytoplasmic proteins, these connections may also play critical roles in junctional assembly. For example, ZO-1 binds directly to α-catenin, and this association is considered an intermediate step in the formation of tight junctions [45]. The residues derived from α-catenin (612–906) in the ELAαC chimera include the ZO-1-binding site (631–906). Thus, ELAαC may use this interaction for tight junction assembly. The residues derived from α-catenin (157–381) in ELAαM do not have the ZO-1-binding site, but they overlap with the reported α-catenin residues 325–360 [52] or 326–377 [53] required for vinculin binding. Thus, ELAαM may use this interaction for tight junction assembly. Another possibility that must be considered is that desmosomal and tight junction assembly requires close cell–cell contacts and the stabilization of junctions mediated by ELAαM and ELAαC, but does not require the molecular interactions mentioned above. However, it has been shown that when the interaction of α-catenin with ZO-1 was specifically prevented by a single amino acid substitution in α-catenin, the integrity of the tight junction, but not that of the adherens junction, was altered [54]. Therefore, we believe that the specific interactions of α-catenin with these proteins are required for junctional assembly.

## Supporting Information

Figure S1 **Confocal imaging of MDCK cells expressing DsRed or DECT.** Cells were stained with anti-β-catenin (A) or anti-plakoglobin (B) antibodies together with DAPI to visualize nuclei. Although a significant part of DECT was detected in cytoplasm together with β-catenin or plakoglobin, a small amount of these proteins was present in the nucleus. In contrast, a significant part of RsRed was detected in nucleus. Bars, 25 µm.
(TIF)
