# Supplementary figures and images for: Cadherin Cytoplasmic Domains Inhibit the Cell Surface Localization of Endogenous E-Cadherin, Blocking Desmosome and Tight Junction Formation and Inducing Cell Dissociation
Source: PLoS One. 2014 Aug 14;9(8):e105313. doi: 10.1371/journal.pone.0105313 (PMC4133371; doi:10.1371/journal.pone.0105313)

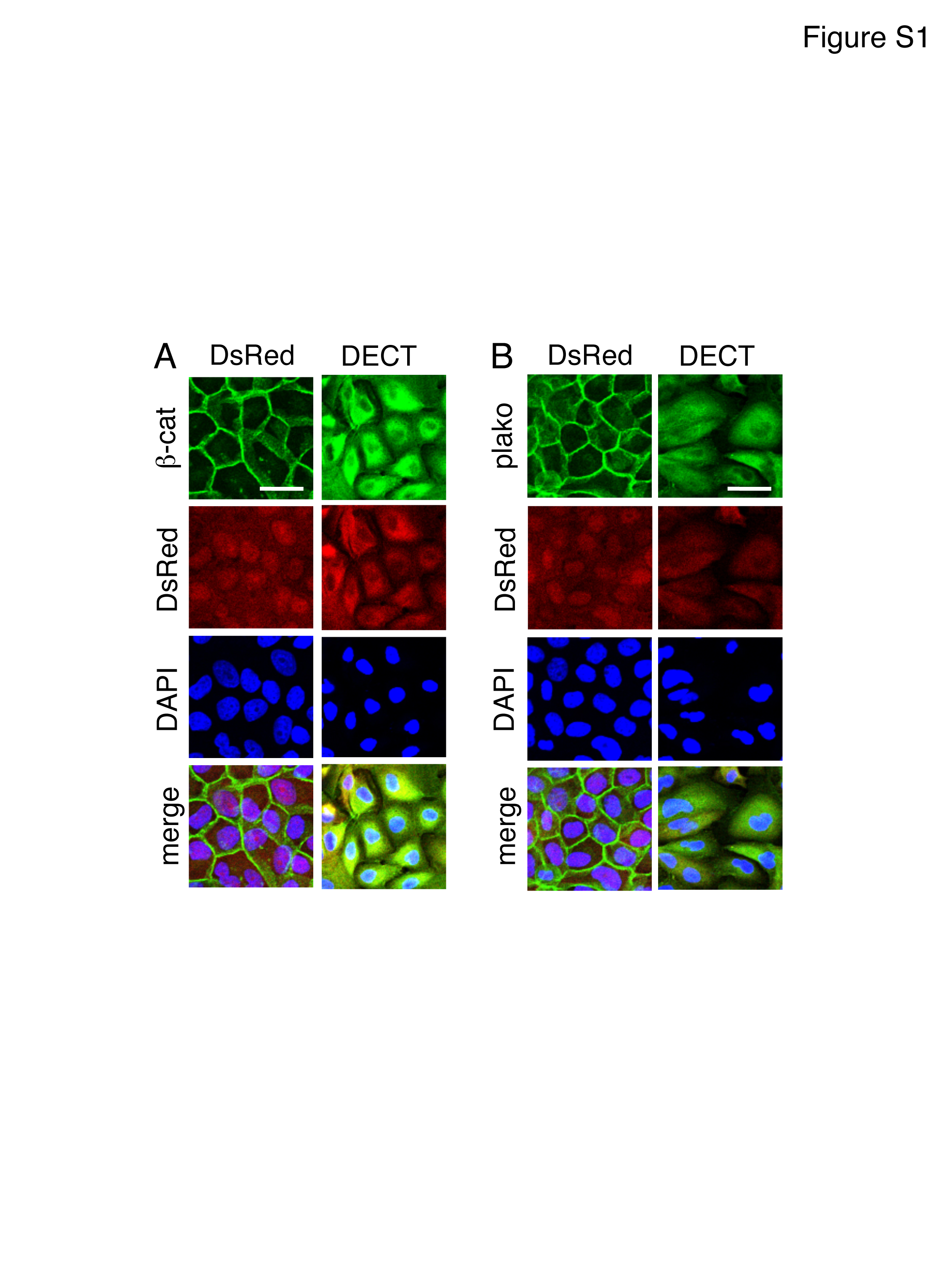

Supplement: Figure S1 — Confocal imaging of MDCK cells expressing DsRed or DECT. Cells were stained with anti-β-catenin (A) or anti-plakoglobin (B) antibodies together with DAPI to visualize nuclei. Although a significant part of DECT was detected in cytoplasm together with β-catenin or plakoglobin, a small amount of these proteins was present in the nucleus. In contrast, a significant part of RsRed was detected in nucleus. Bars, 25 µm. (TIF) [file pone.0105313.s001.tif]
